# Seapolynol Extracted from *Ecklonia cava* Inhibits Adipocyte Differentiation *in Vitro* and Decreases Fat Accumulation *in Vivo*

**DOI:** 10.3390/molecules201219796

**Published:** 2015-12-04

**Authors:** Hui-Jeon Jeon, Hyeon-Son Choi, Yeon-Joo Lee, Ji-Hyun Hwang, Ok-Hwan Lee, Min-Jung Seo, Kui-Jin Kim, Boo-Yong Lee

**Affiliations:** 1 Department of Food Science and Biotechnology, CHA University, Seongnam, Gyeonggi 463-400, Korea; jhj_0324@naver.com (H.-J.J.); eyeonjoo@gmail.com (Y.-J.L.); hwangjh0947@gmail.com (J.-H.H.); mjseo@kirams.re.kr (M.-J.S.); Kuijin.Kim@gmail.com (K.-J.K.); 2 Department of Food Science and Technology, Seoul Women’s University, Hwarang, Nowon, Seoul 139-774, Korea; hschoi@swu.ac.kr; 3 Department of Food Science and Biotechnology, Kangwon National University, Chuncheon 200-701, Korea; loh99@kangwon.ac.kr

**Keywords:** seapolynol, adipogenesis, 3T3-L1, zebrafish, ICR mouse

## Abstract

Seapolynol (SN) is a polyphenol mixture derived from *Ecklonia cava.* We evaluated the effects of SN on lipid accumulation in adipocytes, zebrafish, and mice. SN effectively inhibited lipid accumulation in three experimental models by suppressing adipogenic factors. Triglyceride synthetic enzymes such as diacylglycerol acyltransferase 1 (DGAT1) and GPAT3 were also downregulated by SN. This SN-induced inhibition of adipogenic factors was shown to be due to the regulatory effect of SN on early adipogenic factors; SN downregulated the expression of Krueppel-like factor 4 (KLF4), KLF5, CCAAT-enhancer-binding protein β (C/EBPβ), C/EBPδ, and Protein C-ets-2 (ETS2), while KLF2, an anti-early adipogenic factor, was upregulated by SN. SN-mediated inhibition in early adipogenesis was closely correlated with the inhibition of mitotic clonal expansion via cell cycle arrest. SN inhibited cell cycle progression by suppressing cell cycle regulators, such as cyclin A, cyclinD, and pRb but increased p27, a cell cycle inhibitor. In a mouse study, SN effectively reduced body weight and plasma lipid increases induced by a high-fat diet; triglycerides, total cholesterol, and low-density lipoprotein (LDL) levels were markedly reduced by SN. Moreover, SN remarkably improved high-fat-diet-induced hepatic lipid accumulation. Furthermore, SN activated AMP-activated protein kinase-α (AMPKα), an energy sensor, to suppress acetyl-coA carboxylase (ACC), inhibiting lipid synthesis. Our study suggests that SN may be an edible agent that can play a positive role in prevention of metabolic disorders.

## 1. Introduction

*Ecklonia cava* is an edible brown alga that inhabits the coastal waters of Korea and Japan. It contains many unique polyphenols, especially phlorotannins [1]. Extracts of *Ecklonia cava* have been reported to have various biological activities, such as antioxidant, anticancer, antidiabetes, and antiallergy effects [2,3]. Due to its bioactivities, it has been commercialized and is available as a therapeutic, Seapolynol (SN). The constituents of SN are known to include eckol, dieckol, and bieckol derivatives [1]: dieckol (8.2%), 8,8′-bieckol (2.8%), 2-*O*-(2,4,6-trihydroxyphenyl)-6,6′-bieckol (2.1%), 6,6′-bieckol (1.5%), phlorofurofucoeckol-A (1.4%), eckol (0.6%), 2-phloroeckol (0.4%), 7-phloroeckol (0.4%), and phlorotannin A (0.4%). SN, a mixture of these marine polyphenols, may be a promising edible agent for treating various pathological conditions.

Obesity is the result of excess fat storage, derived from an imbalance in energy. It is a constantly increasing metabolic disease worldwide, leading to atherosclerosis, diabetes mellitus, sleep apnea syndrome, and even some cancers [4]. Treatment of obesity includes control of diets, exercise, and medications. Diet and exercise are important for the management of overweight subjects and the prevention of obesity, but the efficacy of such countermeasures is limited for treating morbid obesity, in particular, those with a body mass index (BMI) ≥ 30 kg/m^2^. There is a continuing need for novel treatments for obesity, and many medications have been developed and marketed [5]. However, current obesity drugs have adverse effects such as dizziness, insomnia, headache, rash, and mild gastrointestinal symptoms [6]. Thus, the development of antiobesity drugs with fewer or no side effects is desirable.

The accumulation of body fat depends on the differentiation status of adipocytes. In cell-based studies, adipocyte differentiation processes are well understood, with hormonal and nutritional regulation [7,8]. The differentiation process begins from a growth-arrested confluent state of the cells, followed by the treatment of a hormone cocktail composed of insulin, dexamethasone, and 3-isobutyl-1-methylxanthine (IBMX). These differentiating cells then restart the cell cycle to increase cell numbers two- to four-fold by mitotic clonal expansion (MCE) [9]. This hyperplasia of adipocytes is accompanied by the expression of early adipogenic transcription factors, such as KLF4 and 5, C/EBPβ, C/EBPδ, and Protein C-ets-2 (ETS2), whereas KLF2 expression is negatively regulated during MCE [10,11,12]. The regulation of early adipogenic factors is closely associated with the expression of late adipogenic factors, such as C/EBPα and peroxisome proliferator-activated receptor gamma (PPARγ). Activation of late adipogenic factors leads to the expression of enzymes involved in triglyceride synthesis, such as lipin1 and DGAT1, promoting lipid accumulation in the adipocyte [13,14,15]. Adipogenic hyperplasia generally involves the activation of cell proliferative signaling pathways, such as the phosphoinositide 3-kinase/protein kinase B (PI3K/AKT) and extracellular signal-regulated kinase (ERK) pathways [16,17]. Moreover, AMPK signaling is involved in the lipid biosynthetic pathway, controlling energy metabolism in adipocytes [18]. Activation of AMPK reduces fatty acid synthesis by deactivating acetyl-coA carboxylase (ACC), a major fatty acid synthetic enzyme [19].

In recent years, the zebrafish has been identified as a useful animal model for the study of various human diseases. Benefits of the zebrafish model include its short generation time, abundant embryos, and lower costs compared with other animal models [20]. Zebrafish also provide a transparent body for easier observation of some phenotypes. In particular, the programs for lipid accumulation of zebrafish are similar to those of mammals [21]. Accordingly, research into obesity has been conducted using the zebrafish model [22]. As the mouse is a well-established animal model of obesity, a comparative study of both models would enhance the reliability of any results.

Recently, SN, a mixture of edible marine polyphenols, has been reported to have antihyperlipidemic effects [23]. However, the mechanistic understanding of the inhibitory effect of SN is limited. In this study, we investigated the inhibitory effects of SN on lipid accumulation using various models: 3T3-L1 cells, zebrafish, and mice. Additionally, the mechanistic relevance of cell cycle state and signaling in early adipogenesis was examined.

## 2. Results

### 2.1. Effects of SN on Cytotoxicity, Lipid Accumulation, Adipogenic Factors, and Triglyceride Synthetic Enzymes during Adipocyte Differentiation

**Figure 1:**
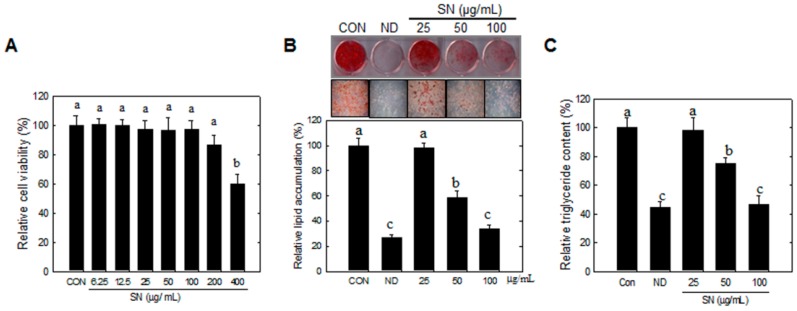
Effects of SN on lipid and triglyceride in adipocyte. Differentiation was induced by incubation with a hormone cocktail in the presence or absence of SN for eight days. (**A**) Cytotoxicity of SN treatment (**B**) After eight days, the cells were stained with Oil red O, which was subsequently eluted in isopropanol and its optical density at 490 nm was measured; (**C**) Triglyceride (TG) accumulation was determined using a TG assay kit (Zen Bio, Inc., Research Triangle Park, NC, USA). CON: control, SN: seapolynol, ND: non-differentiated 3T3-L1. Data are presented as means ± SD from three replicates. Means not designated by a common superscript are significantly different (*p* < 0.05).

Our data showed that SN did not significantly affect cell viability at doses lower than 200 µg/mL (Figure 1A). Cells showed no change in morphology in a microscopic analysis (data not shown). Oil red O staining showed that SN inhibited lipid accumulation during adipocyte differentiation in a dose-dependent manner (Figure 1B). A 100 µg/mL dose of SN led to a marked (65%) reduction in lipid accumulation, compared with the control. The SN-induced inhibition of lipid accumulation was also correlated with a reduction in triglyceride levels (Figure 1C).

**Figure 2:**
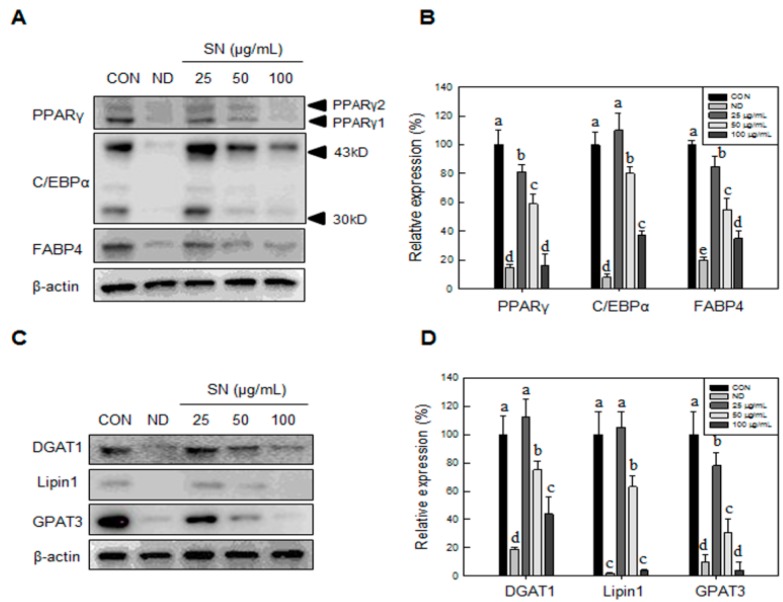
Effects of SN on the expression of adipogenic factors and triglyceride synthetic enzymes in adipocyte. (**A**) Late adipogenic factors were assessed by Western blotting; and (**B**) levels of triglyceride synthetic enzymes were quantified using the ImageJ software; (**C**) Fatty acid synthase enzymes were assessed by Western blotting; and (**D**) levels of triglyceride synthetic enzymes were quantified using the ImageJ software. Differentiated or undifferentiated cells were harvested at day 6 for extraction of proteins, which were subjected to Western blotting. CON: control, SN: seapolynol, ND: non-differentiated 3T3-L1. Data are presented as means ± SD from three replicates. Means not designated by a common superscript are significantly different (*p* < 0.05).

This SN-induced reduction of lipid storage was shown to be associated with downregulation of adipogenic factors (Figure 2A) and triglyceride synthetic enzymes (Figure 2B). SN at 100 µg/mL inhibited the expression of adipogenic factors by 60%–80% (Figure 1B). Triglyceride synthetic enzymes, including Lipin1 and GPAT3 (Figure 2C,D), were greatly reduced, by over 80%, in their expression level. These results indicated that SN inhibited triglyceride synthesis by suppressing the triglyceride synthetic pathway and expression of adipogenic factors during adipocyte differentiation.

### 2.2. Effect of SN on Early Adipogenic Stages

**Figure 3:**
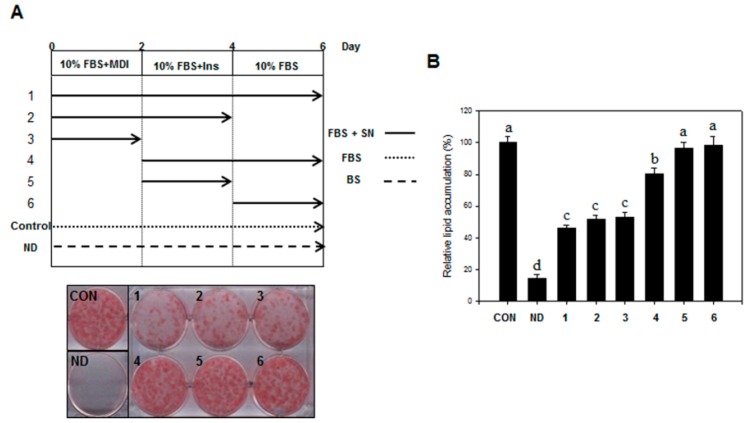
Effect of SN on mitotic clonal expansion during the early stages of adipogenesis. (**A**) Differentiation was induced by incubation with a hormone cocktail in the presence or absence of SN on days 0–2, 0–4, 0–6, 2–4, 2–6, or 4–6. After six days, the cells were stained with Oil red O, which was subsequently eluted in isopropanol and (**B**) its optical density at 490 nm was measured. CON: control, SN: seapolynol, ND: non-differentiated 3T3-L1. INS: insulin, BM: growth medium, MDI: hormonal cocktail, FBS: differentiation medium, DMSO: dimethyl sulfoxide. Data are presented as means ± SD from three replicate experiments. Means not designated by a common superscript are significantly different (*p* < 0.05).

We examined the adipogenic stage that was inhibited by SN. Treatment with SN (100 µg/mL) during the early adipogenic stage (days 0–2) resulted in significant inhibition of lipid accumulation (Figure 3A), compared with the control. SN treatments during days 0–6, 0–4, and 0–2 resulted in an inhibition of lipid accumulation by ~50%, compared with the control (Figure 3B). However, treatment with SN after day 2 resulted in only a weak inhibitory effect (20%) on lipid accumulation, and no inhibitory effect was observed when treatment was performed after day 4. These results demonstrated that SN primarily inhibited lipid accumulation in the early adipogenic stages.

Next, we examined the effect of SN on the mRNA levels of transcription factors responsible for early adipogenesis. The mRNA levels of the early adipogenic factors KLF4, KLF5, C/EBPβ, C/EBPδ, and ETS2 were significantly reduced by SN treatment (100 µg/mL).

**Figure 4:**
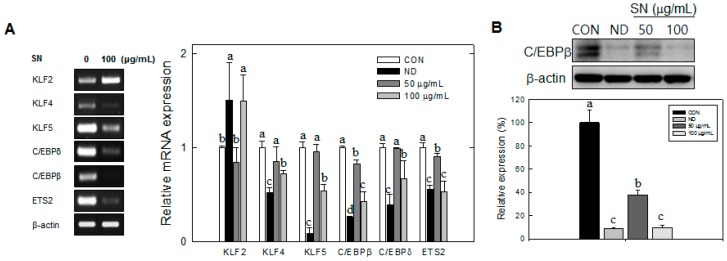
SN regulates mitotic clonal expansion associated genes during the early stages of adipogenesis. (**A**) Mitotic clonal expansion associated genes were assessed by semi-quantitative RT-PCR or qRT-PCR. The levels of gene expression were quantified using the ImageJ software; (**B**) C/EBPβ was assessed by Western blot. The level of protein expression was quantified using the ImageJ software. CON: control, SN: seapolynol, ND: non-differentiated 3T3-L1. Data are presented as means ± SD from three replicate experiments. Means not designated by a common superscript are significantly different (*p* < 0.05).

In particular, SN treatment showed a dose-dependent reduction of C/EBPβ expression at the mRNA (Figure 4A) and protein levels (Figure 4B). KLF4 was decreased by 20% and 30% with 50 and 100 µg/mL doses, respectively. KLF5 and C/EBPβ and mRNA levels were decreased by 60% or more, compared with the control with the 100 µg/mL dose, but those of KLF4, KLF5, and C/EBPδ did not show a significant difference *vs.* the control group with a 50 µg/mL dose. Conversely, the mRNA level of KLF2, a negative regulator of adipogenesis, was increased by 50% with the 100 µg/mL dose. These results indicated that SN inhibited adipocyte differentiation via regulation of early adipogenic factors.

### 2.3. Effect of SN on Mitotic Clonal Expansion (MCE)

**Figure 5:**
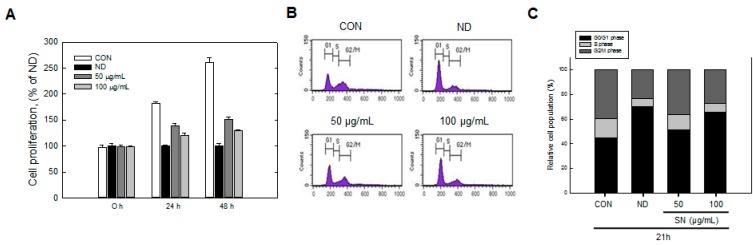
Effects of SN on cell cycle progression of 3T3-L1 cells during differentiation. (**A**) After differentiation for 24 or 48 h, the number of cells stained with trypan blue was determined using a hemocytometer; (**B**) 3T3-L1 cells, in which differentiation was induced by treatment with a hormone cocktail with or without SN for 24 h, were harvested, fixed with 70% ethanol and stained with propidium iodide. Stained DNA was analyzed using a flow cytometer; (**C**) The cell population at each stage of the cell cycle was determined using the BD CellQuest Pro software (version 3.01, Becton Dickinson, San Jose, CA, USA). CON: control, SN: seapolynol, ND: non-differentiated 3T3-L1, G1: growth 1 phase of the cell cycle, S: synthesis phase of the cell cycle, G2/M: growth 2 and mitosis phase of the cell cycle.

The early adipogenic phase of adipocytes includes the MCE stage, during which the number of adipocytes increases markedly (Figure 5A).

Trypan blue counting showed that SN inhibited cell proliferation in a dose-dependent manner during this stage. The normal control (no treatment) group showed an over twofold increase in cell numbers after 48 h. However, SN treatment effectively inhibited this increase, and cell numbers with 100 µg/mL of SN were reduced by ~85% compared with the control group. This result demonstrated that SN inhibited adipogenesis in the MCE phase.

### 2.4. Effect of SN on Cell Cycle Progression

**Figure 6:**
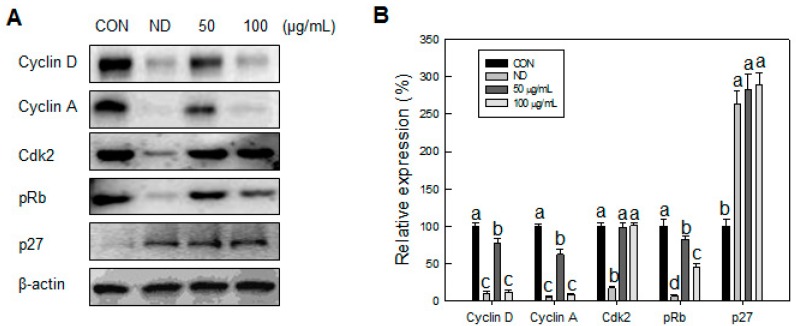
Effects of SN on cell cycle progression of 3T3-L1 cells during differentiation. (**A**) After differentiation. After 24 h of differentiation, cells were lysed for analysis of cyclins and cdk2 by Western blotting and (**B**) levels of cell cycle regulatory genes were quantified using the ImageJ software. CON: control, SN: seapolynol, ND: non-differentiated 3T3-L1. Data are presented as means ± SD from three replicate experiments. Means not designated by a common superscript are significantly different (*p* < 0.05).

We identified an inhibitory effect of SN on cell proliferation in the MCE stage. Accordingly, we next measured the effect of SN on the cell cycle during the early adipogenic MCE stage. Undifferentiated cells did not progress in the cell cycle, due to contact inhibition, with most of the cell population (~70%) in the G0/G1 phase (Figure 5B,C). Normal cell cycle progression occurred, from the G0/G1 phase to the S and G2/M phases, when adipogenic inducers were added. The cell population in the G0/G1 phase decreased to 44%, whereas the cell population in the S phase increased from 8% to 16%, and that in M phase increased from 22% to 40%, compared with undifferentiated cells. However, SN treatment inhibited cell cycle progression in a dose-dependent manner. SN treatment reduced the S phase cell population from 16% to 14% and 9% following 50 and 100 µg/mL doses, respectively. M phase also decreased, to 28%, and the proportion of G1 phase cells was increased from 44% to 65% with 100 µg/mL SN. These results suggest that SN inhibited cell cycle progression. This SN-induced inhibition was supported by the regulation of cell-cycle regulators. The expression of Cyclin D and pRb, which regulate the G1 phase, were dose-dependently decreased by SN treatment (Figure 6). Additionally, cyclin A, a major cell-cycle regulator in the S phase, was downregulated by SN treatment. In contrast, expression of cyclin-dependent kinase 2 (Cdk2), a cyclin partner, was unaffected by SN. However, a negative regulator, p27 was increased greatly by SN treatment. These results showed that the major cell cycle regulatory proteins include cyclines were suppressed by SN treatment in 3T3-L1 adipocyte.

### 2.5. Effects of SN on Cell Proliferation and Lipid Metabolism Signaling Processes

**Figure 7:**
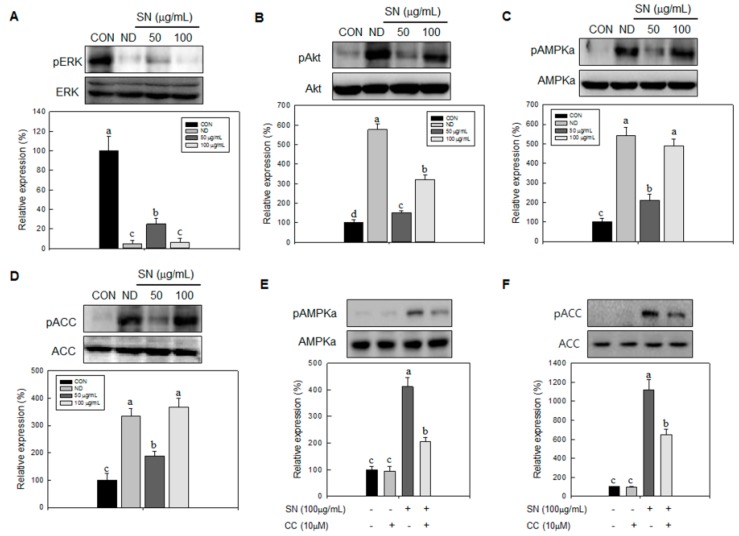
Effects of SN on signaling pathways. 3T3-L1 preadipocyte was treated with and without SN during adipocyte differentiation. (**A**–**C**) Expression levels of signaling factors were analyzed in lysates harvested after 1 or 2 h of differentiation, measured by immunoblotting, and quantified using the ImageJ software; (**D**–**F**) An inhibitor (Compound **C**, 10 µM) of the AMPK signaling pathway was added to measure the dependent effect of SN (100 µg/mL) on AMPKα signaling. CON: control, SN: seapolynol, ND: non-differentiated 3T3-L1, CC: Compound **C**. Data are presented as means ± SD from three replicate experiments. Means not designated by a common superscript are significantly different (*p* < 0.05).

Next, we determined the effects of SN on signaling processes responsible for cell proliferation and lipid synthesis. SN effectively inhibited the activation of Akt and ERK, by suppressing their phosphorylation (Figure 7A,B). These results indicated that SN-induced inhibition of cell proliferation of adipocytes was achieved by the suppression of Akt and ERK signaling. However, SN treatment led to the activation of AMPKα, a key energy sensor in cell metabolism, by increasing its phosphorylation level (Figure 7C). Additionally, its target protein, ACC, a major enzyme in lipid synthesis, was repressed by SN treatment (Figure 7D). These data suggest that SN inhibits lipid accumulation by activating AMPKα signaling. We further determined whether SN-induced inhibition of lipid synthetic signaling was mediated by activation of AMPKα signaling. A specific AMPKα inhibitor, compound **C**, suppressed SN-induced phosphorylation of AMPKα and ACC, even if their basal phosphorylation was not significantly affected by SN treatment (Figure 7E,F). These results indicated that the SN-induced repression of ACC was mediated via AMPKα activation.

### 2.6. Effects of SN on Lipid Accumulation in Zebrafish

**Figure 8:**
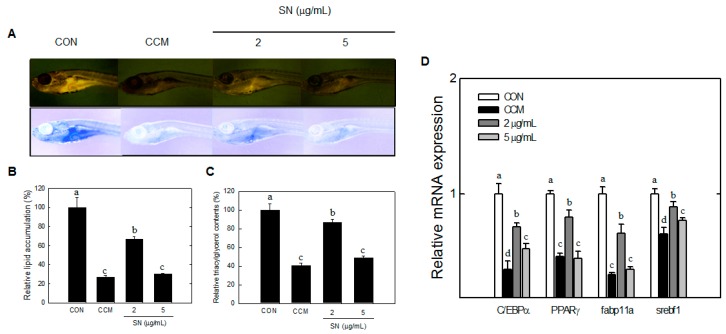
Effects of SN on lipid accumulation in zebrafish. (**A**) Live larvae were visualized under a fluorescence microscope, and the image color was inverted; (**B**) Fluorescence microscopy image were quantified by using ImageJ software; (**C**) Triglyceride levels in zebrafish were determined using a TG Assay Kit (Zen Bio, Inc.); (**D**) mRNA expression of adipogenic factors in zebrafish was quantified by qRT-PCR. CON: high fat diet, CCM: curcumin, SN: seapolynol. Data are presented as means ± SD of 20 zebrafish. All data are representative of three replicates. Means not designated by a common superscript are significantly different (*p* < 0.05).

The effects of SN on early events in lipid accumulation were assessed in zebrafish by Nile red staining. SN treatment significantly decreased the level of fluorescence, which reflects accumulated lipids (Figure 8A). Quantitative fluorescence analysis indicated that treatment with 5 µg/mL SN resulted in a >70% decrease in Nile red staining of the fish, compared with the control (Figure 8B).

Moreover, curcumin, is an antiadipogenic dietary compound [24], showed ~75% inhibition of Nile red staining. This result was supported by analyses of triglycerides and adipogenic factor. Triglyceride content in zebrafish was decreased by 50% with the 5 µg/mL SN dose (Figure 8C). Major adipogenic factors, PPARγ, C/EBPα, SREBF-1, and fabp11a, were significantly reduced by SN treatment (Figure 8D). These results showed that SN inhibited the accumulation of triacylglycerols via downregulation of adipogenic factors in zebrafish.

### 2.7. Effects of SN on Lipid Accumulation in the Mouse

**Figure 9:**
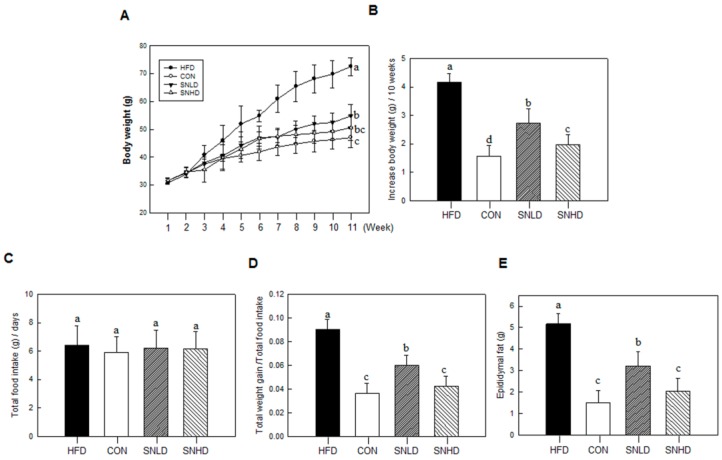
Effects of SN on physical changes in high-fat-diet-induced obese mouse model. (**A**) Growth curve; (**B**) Body weight gain; (**C**) Total food intake; (**D**) Body weight gain normalized by food intake; (**E**) Epidymal fat. CON: normal diet, HFD: high fat diet, SN: seapolynol, SNLD: seapolynol low dose, SNHD: seapolynol high dose. Data are presented as means ± SD of eight mice. Means not designated by a common superscript are significantly different (*p* < 0.05).

SN supplementation (SNLD and SNHD) for 11 weeks significantly reduced the final body weight of mice fed a high-fat diet (HFD); SNLD and SNHD decreased by 25% and 50%, respectively, *vs.* the HFD group (Figure 9A,B). No group showed any significant change in food intake (Figure 9C).

**Figure 10:**
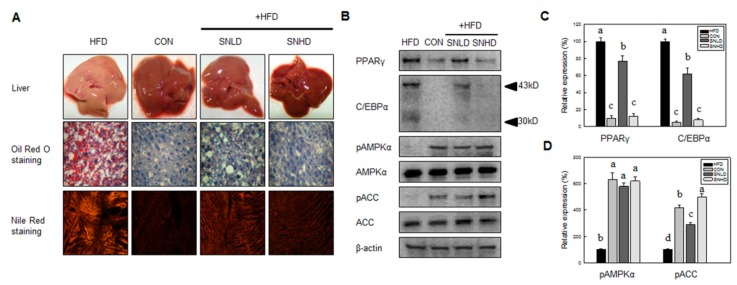
Effects of SN on the hisotophathological and proteomic analysis of hepatic tissue in high-fat-diet-induced obese mouse model. (**A**) Histopathological analysis of hepatic tissue; (**B**) Ptoteomic analysis of Body weight gain; (**C**) Total food intake; (**D**) Body weight gain normalized by food intake; (**E**) Western blot analysis of PPARγ, C/EBPα, pAMPKα, AMPKα, pACC, ACC and β-actin in hepatic tissue. CON: normal diet, HFD: high fat diet, SN: seapolynol. Data are presented as means ± SD of eight mice. Means not designated by a common superscript are significantly different (*p* < 0.05).

The food efficiency ratio of SNLD- and SNHD-fed mice was reduced by 33% and 65%, compared with that of HFD-fed mice, respectively (Figure 9D). This result is consistent with the reductions (40% and 60%) in epididymal fat observed in SNLD and SNHD mice (Figure 9E). Additionally, Oil Red O and Nile red staining of liver indicated that liver status was effectively improved by SNLD and SNHD treatments. The HFD group resulted in a fatty liver, with a white color, while normal diet-fed mice had relatively healthy, dark-pink livers (Figure 10A). However, SN treatments effectively reversed the fatty liver induced by HFD. These results indicated that SN inhibited the development of a fatty liver in response to a HFD.

HFD-induced elevations in total triglycerides, total cholesterol, and LDL cholesterol were significantly reduced by SN treatments (Table 1). In particular, the LDL cholesterol level was decreased by 66% compared with that of the HFD-fed group, showing a similar level to the normal diet group. Triglycerides and total cholesterol levels were also decreased by 40% and 32%, respectively, compared with the control group. However, the HDL cholesterol, plasma glucose, and insulin levels were not significantly affected by SN supplementation.

Additionally, the protein levels of major adipogenic factors, such as PPARγ and C/EBPα, were effectively inhibited by SN supplementation (Figure 10B,C), as seen in the cell line study. Conversely, the energy sensor AMPKα and its downstream protein ACC were activated by SN supplementation (Figure 10D), correlating with the SN-induced activation of AMPKα and ACC in adipocytes. Our data showed that the SN-mediated inhibition of lipid accumulation in HFD-fed mice was associated with the downregulation of adipogenic factors and activation of AMPKα signaling.

**Table 1:** Mouse plasma lipid, glucose, and insulin levels.

	Groups	HFD Only	Normal Diet	HFD + SNLD	HFD + SNHD
Variables					
Triglyceride (mg/dL)		166.8 ± 9.5 ^a^	131.3 ± 5.9 ^b^	123.7 ± 7.3 ^b^	100.4 ± 7.9 ^c^
Total Cholesterol (mg/dL)		143.6 ± 5.6 ^a^	106.7 ± 9.7 ^c^	120.9 ± 5.3 ^b^	111.7 ± 9.8 ^c^
HDL-Cholesterol (mg/dL)		90.1 ± 7.5 ^a^	78.5 ± 12.1 ^a^	93.1 ± 2.1 ^a^	98.6 ± 6.2 ^a^
LDL-Cholesterol (mg/dL)		40.1 ± 5.5 ^a^	21.9 ± 2.1 ^b^	23.1 ± 2.5 ^b^	13 ± 4.1 ^c^
Glucose (mg/dL)		117.5 ± 12.1 ^a^	109 ± 12.4 ^a^	109.8 ± 10.4 ^a^	112.8 ± 9.7 ^a^
Insulin (ng/dL)		1.1 ± 0.4 ^a^	0.9 ± 0.3 ^a^	1.0 ± 0.3 ^a^	1.1 ± 0.3 ^a^

Means not designated by a common superscript are significantly different (*p* < 0.05).

## 3. Discussion

Our study showed that SN inhibited lipid accumulation in 3T3-L1 cells and high-fat-diet-fed zebrafish and mice. Furthermore, we examined the underlying mechanisms of the SN-induced antiadipogenic effect including inhibition of MCE via cell cycle arrest, and activation of AMPKα signaling. *Ecklonia cava* has been shown to inhibit adipogenesis in cell lines. Several polyphenols (dieckol, phloroeckol, dioxinodehydroeckol) derived from *Ecklonia cava* were shown to have antiadipogenic effects in adipocytes [1,16]. A clinical study on *Ecklonia cava* polyphenols was also performed in overweight subjects. In particular, the most abundant *Ecklonia cava* polyphenol, dieckol [3,4], has been identified to have antiadipogenic effects in cells and animal models [17]. SN, a standardized extract of *Ecklonia cava*, includes active compounds including the polyphenols mentioned above, and is commercially available; SN contains dieckol, eckol, and bieckol derivatives [1]. Recent studies have shown the biological effects of this extract. *Ecklonia cava* extract or *Ecklonia cava-*derived polyphenols have been reported to have antioxidant effects [2,25]. Kang *et al.*, showed the potential of *Ecklonia cava* polyphenols as chemopreventive agents [25], and the antioxidant and anti-inflammatory effects of *Ecklonia cava* in osteoarthritis were evaluated. *Ecklonia cava* extract was shown to have antibacterial effects on antibiotic-resistant food-borne pathogens [26]. Additionally, Heo *et al.*, showed protective effects of *Ecklonia cava* phlorotannins against photo-oxidative stress from UV-B radiation [27]. Shibata *et al.*, reported the inhibitory effect of *Ecklonia cava* phlorotannins on pro-inflammatory responses [28]. In particular, an antiadipogenic effect of SN has been reported recently [23]. However, the mechanisms underlying the antiadipogenic effects of SN should be investigated. The current study is the first to show the antiadipogenic effect of SN in two animal models with early adipogenic and signaling mechanisms, in which zebrafish was used to show the effects of SN in early adipogenesis. Lipid accumulation in zebrafish is known to be detectable from 15 dpf [29]. Zebrafish were grown for 17–20 dpf to show early fat accumulation by Nile-red staining; SN inhibited fat accumulation in zebrafish from an early stage.

In addition, the diet induced obese mice has been used here were 5 weeks old, fed on HFD for 10 weeks. The HFD mice caused a marked increase the lipid accumulation in the liver or TG and total cholesterol in the blood compared to control diet mice, while SN dramatically repressed the lipid accumulation in the liver or TG and total cholesterol in the blood. In addition, SN significantly decreased HFD-induced abnormal expression of adipogenic and fatty liver associated genes. On the other hand, the high levels of glucose and insulin concentration are generally occurring in HFD-induced obese mice [30]. However, we did not observed the sign of hyperglycemia and hyperinsulinemia in HFD-induced obese mice compared to the control diet group in this study.

The afore-mentioned evidence prompt us to speculate that some of the factors such as mouse strain, ages, diet composition or duration of HFD may affect to develop the intermediate stage obesity between obesity and type 2 diabetes. This model may occur the some of genetic changes in the tissues due to the HFD consumption in mice, but it has not yet appeared the hyperglycemia and hyperinsulinemia. However, additional animal study should be analyzed in further studies.

Our data showed that SN induced inhibition in early adipogenesis via regulating early adipogenic factors, such as KLF2, KLF4, and C/EBPβ. SN-mediated inhibition of early adipogenesis was associated with the inhibition of MCE via cell cycle arrest, and the inhibition of ERK and Akt signaling was underlined as a reason for the SN-induced inhibition of early adipogenesis. Recent studies have shown that several phytochemicals induce inhibition of MCE via cell cycle arrest as one of the major mechanisms of an antiadipogenic effect in 3T3-L1 cells [31]. In particular, our data showed that SN activates AMPKα, a sensor in energy metabolism, and ACC, a major enzyme in fatty acid synthesis, in both adipocytes and a mouse model. Such AMPKα activation was also shown previously in a study of dieckol [32]. Accordingly, the SN-induced activation of AMPKα is likely due to dieckol, a major component of SN. However, the other compounds in SN should also be investigated. The SN-mediated repression of ACC could contribute to the suppression of TG accumulation in adipocytes and mice models. Our inhibitor study showed SN-induced repression of ACC was mediated via activation of AMPKα signaling.

Our study showed that SN supplementation effectively improved the plasma lipid status of mice fed HFD, as was seen with dieckol [32]. However, SN was superior to dieckol in terms of the suppression of lipid accumulation and cholesterol levels. SN decreased triglyceride levels by 40%—while dieckol reduced them by only ~10%—in mice fed, an HFD. LDL cholesterol levels were reduced by 66% in SN-supplemented HFD-fed mice, while dieckol supplementation showed a 54% reduction in LDL cholesterol [32]. This result was supported by the study of Yeo *et al.*, [23] which showed SN is more effective than dieckol on inhibition of lipid accumulation and weight increase. Given that dieckol comprises 8% of SN [1], other SN-derived compounds likely contribute to the difference in effects on lipid accumulation and cholesterol levels between SN and dieckol. SN showed significant effects in decreasing triglyceride and cholesterol levels in HFD-fed mice.

The reductions in triglycerides and lipid accumulation by SN are supported by its suppression of adipogenic factors. However, the SN-induced reduction of (LDL) cholesterol level was not fully addressed in this study. In particular, downstream targets of AMPKα include various factors—including HMGCoA and SREBP1, which are responsible for cholesterol metabolism [33]. Although SREBF-1 was shown to be regulated by SN in zebrafish, other cholesterol-related factors should be examined. AMPKα also regulates carbohydrate metabolism via factors such as ChREBP, but glucose and insulin levels were unaffected by SN, suggesting that SN does not greatly affect carbohydrate metabolism.

AMPKα is known to be associated with factors related to the energy expenditure, such as the NAD-dependent protein deacetylase sirtuin 1 (SIRT1) and peroxisome proliferator-activated receptor-γ coactivator 1α (PGC1α) [29]. These signaling processes are known to activate thermogenic responses, such as heat production with upregulation of uncoupling proteins [29]. A recent study showed that a phytochemical, indole-3-cabinol, directly activated SIRT1 via binding in 3T3-L1 cells [34]. Thus, the roles of SN and SN-derived compounds should be investigated in thermogenic signaling in future studies.

We also used the zebrafish model, and showed SN-mediated inhibition in early adipogenesis and lipid accumulation. Zebrafish provides various advantages for *in vivo* research but also has several limitations. Full sets of antibodies for lipid metabolism studies have not yet been established. Further studies should include data for the antiadipogenic effects of SN in protein levels. Finally, our data demonstrated that SN effectively suppressed HFD-induced lipid accumulation in cell line, zebrafish, and mouse models.

*Ecklonia cava* contains various polyphenols, known as phlorotannins. Phloroeckol, dioxinodehydroeckol, and dieckol have been shown to have antiadipogenic activities in adipocytes and animals [23], and dieckol, a major compound of SN, could give an important contribution to antiadipogenic effects of SN. However, the effect may be derived from the synergistic or combinational effects of constituents in SN. Since other *Ecklonia cava*-derived polyphenols, which may also have antiadipogenic effects, have yet to be fully investigated, further study should be performed on SN-derived compounds, additionally, comparative and combinational studies on SN-derived compounds would be executed in the future.

## 4. Experimental Section

### 4.1. Materials

Seapolynol was obtained from Botamedi (Jeju, Korea). Dulbecco’s modified Eagle’s medium (DMEM), bovine calf serum (BCS), fetal bovine serum (FBS), penicillin-streptomycin (P/S), phosphate-buffered saline (PBS), and trypsin-EDTA were obtained from Gibco (Gaithersburg, MD, USA). Dexamethasone (DEX), IBMX, insulin, Oil red O, and nitrobluetetrazolium (NBT) were purchased from Sigma-Aldrich (St. Louis, MO, USA). Antibodies against pRB, Akt, pAkt, ERK, pERK, C/EBPβ, AMPKα, pAMPKα, ACC, pACC, and β‑actin were purchased from Cell Signaling Technology (Danvers, MA, USA). Antibodies against PPARγ, C/EBPα, FABP4, GPAT3, Lipin1, DGAT1, Cyclin A, Cyclin D, Cdk2, and P27 were purchased from Santa Cruz Biotechnology, Inc. (Santa Cruz, CA, USA). Cell lysis buffer and phosphatase inhibitor cocktails I and II were purchased from Sigma. Dorsomorphin dihydrochloride (compound **C**) was purchased from Santa Cruz Biotechnology (Dallas, TX, USA). All other chemicals were purchased from Sigma.

### 4.2. Cell Culture

3T3-L1 preadipocytes were obtained from the American Type Culture Collection (ATCC, CL-173, Manassas, VA, USA). The preadipocytes were plated and cultured in DMEM medium containing 3.7 g/L sodium bicarbonate, 1% P/S, and 10% BCS at 37 °C in 5% CO_2_. Adipocyte differentiation was induced by treating two-day post-confluent cells with 10% FBS and a hormone mixture (MDI), consisting of 0.5 mM IBMX, 1.0 μM DEX, and 1.67 μM insulin (day 0). The medium was replaced with DMEM containing 1.67 μM insulin and 10% FBS for two additional days (day 2). This medium was then replenished with DMEM containing 10% FBS every other day until the indicated time (days 6 to 12). SN (25, 50, and 100 µg/mL) was dissolved in DMSO, the final concentration of DMSO was set to 0.25% in all experiments.

### 4.3. Cell Viability

3T3-L1 preadipocytes or confluent cells were treated with SN (0–400 μg/mL), and incubated for 48 h. XTT (2,3-Bis(2-methoxy-4-nitro-5-sulfophenyl)-2Htetrazolium-5-carbox-anilide) reagent and PMS (*N*-methyl dibenzopyrazine methyl sulfate) were added to the culture and incubated for 2 h at 37 °C. Produced soluble formazan salt in media was measured at 450 nm against 690 nm. Cell viability (%) was expressed as the ratio of viable cells compared to the non-treated cells.

### 4.4. Trypan Blue Assay

Post-confluent 3T3-L1 cells were induced to differentiate with MDI cocktail for two days in the presence or absence of SN. Cells were trypsinized after two washes with PBS. Trypan blue dye (0.5%, Sigma-Aldrich) was added to the cells for 3 min. Stained viable cells were counted under a microscope.

### 4.5. Oil Red O Staining and Lipid Quantification

Differentiated 3T3-L1 cells or liver tissue samples were fixed with 4% formaldehyde at 4 °C for 1 h after washing with PBS. After washing fixed cells with phosphate-buffered saline, cells were stained with 0.5% Oil red O in 60:40 (*v*/*v*) isopropanol/H2O for 2 h at room temperature, and then washed four times with distilled water. The extent of lipid storage was observed microscopically and photographed. Isopropanol (100%) was used to elute Oil red O dye for determination of optical density at 490 nm. Accumulated triglycerides were measured using a Total Triglyceride Assay Kit (Zen-Bio, Inc., Research Triangle Park, NC, USA).

### 4.6. Western Blotting

Proteins extracted from differentiated or undifferentiated and mouse liver tissues were resolved by SDS-PAGE in 8%–12% polyacrylamide gels before they transferred onto PVDF (polyvinylidene fluoride) membrane. An immunoblot analysis was conducted with the commercial antibodies indicated. Secondary antibodies conjugated with horseradish peroxidase (1:1000) were applied for 1 h. Bands were visualized by enhanced chemiluminescence, and detected using the LAS-4000 imaging software (Fuji, New York, NY, USA).

### 4.7. Semi-Quantitative RT-PCR or Real-Time RT-PCR (qRT-PCR)

Total RNA was isolated from 3T3-L1 cells using the TRIzol reagent (Invitrogen, Carlsbad, CA, USA. RNA samples with an OD_260_/OD_280_ ratio higher than 2.0 were used for semi-quantitative RT-PCR. One microgram of total RNA was used to produce cDNA with a RT-PCR system.

For the qRT-PCR, cells, mouse tissues and zebrafishes were washed with phosphate-buffered saline. Then, total RNA was extracted from differentiated (day 6 or 8) or undifferentiated adipocytes, zebrafish, and mouse liver tissues using the TRIzol reagent (Invitrogen, Carlsbad, CA, USA), according to the manufacturer’s protocol. cDNA was produced from 1 µg of total RNA using a Maxime RT PreMix Kit (iNtRON Biotechnology, Inc., Seoul, Korea). cDNA was amplified using SYBR Green 2× master mix kit (M-Biotech. Inc., Salt Lake City, UT, USA) and the specific primers listed in Table 2. The reaction was conducted with a CFX96 Real-Time system and c1000 thermal cycler (Bio Rad, Inc., Hercules, CA, USA) and subjected to PCR analysis at 40 cycles. Quantification analysis was generated using the CFX Manager software (Bio Rad, Inc.).

**Table 2:** Primer sequences.

Name	Forward (5′ to 3′)	Reverse (5′ to 3′)
PPARγ	CTGTGAGACCAACAGCCTGA	AATGCGAGTGGTCTTCCATC
C/EBPα	TGAAGGAACTTGAAGCACAA	TCAGAGCAAAACCAAAACAA
aP2	TCACCTGGAAGACAGCTCCT	AATCCCCATTTACGCTGATG
C/EBPβ	CAAGCTGAGCGACGAGTACA	AGCTGCTCCACCTTCTTCTG
C/EBPδ	AGAAGCTGGTGGAGTTGTCG	CGCAGGTCCCAAAGAAACTA
Krox20	AGTTGGGTCTCCAGGTTGTG	GGAGATCCAGGGGTCTCTTC
KLF4	CTGAACAGCAGGGACTGTCA	GTGTGGGTGGCTGTTCTTTT
KLF5	ACGTACACCATGCCAAGTCA	GTGGGAGAGTTGGCGAATTA
KLF2	GCCTGTGGGTTCGCTATAAA	AAGGAATGGTCAGCCACATC
ETS2	CGCCCCAAGATATTCTGTGT	TGGAAGATCCCTCCTGATTG
β-actin	AGCCATGTACGTAGCCATCC	CTCTCAGCTGTGGTGGTGAA
PPARγ (D)	CAGTTTGCAGAGAACAGCGT	GGCTCTTCTTGTGTATGCGG
C/EBPα (D)	ATCAGCGCCTACATTGATCC	TTGCTTGGCTGTCGTAGATG
aP2 (D)	GCAAACTTGTGCAGAAACA	GAACTGAGCCTGGCATCTTC
β-actin (D)	CTCTTCCAGCCTTCCTTCCT	CTTCTGCATACGGTCAGCAA

### 4.8. Analysis of Cell Cycle Progression

Cell cycle progression of cultured cells was determined by flow cytometry. Post-confluent preadipocytes were treated with MDI in the presence or absence of SN (50 or 100 µg/mL) for 24 h. Collected cells were fixed with 70% ethanol for 2 h at 4 °C, washed with PBS, and centrifuged (1000× *g*) to remove ethanol. For staining the DNA, the resulting pellet was resuspended in 40 µg/mL propidium iodine solution containing 1 mg/mL RNase A at 37 °C for 30 min. The cell cycle progression of samples (10,000 cells per experiment) was analyzed using a BD FACSCalibur flow cytometer (Becton Dickinson, CA, USA), according to the manufacturer’s instructions.

### 4.9. Animal Care and Experimental Protocol

All experimental mice were housed in a specific pathogen-free facility at CHA University, Seongnam, Korea. The project was approved by the Institutional Animal Care and Use Committee of CHA University (IACUC140001). In total, 40 male Imprinting Control Region (ICR) mice (5 weeks old) were purchased from Joong-Ang Experimental Animal Co. (Seoul, Korea) and were housed in a pathogen-free facility under the following conditions: 21 ± 2.0 °C, 50% ± 5% relative humidity, and 12/12-h light/dark cycle. All the mice were fed rodent chow and tap water *ad libitum* for one week prior to their division into the following experimental groups (*n* = 10 per group): normal diet, HFD, and SN-supplemented diets (SNLD; 30 mg/kgBW/day and SNHD; 75 mg/kgBW/day). The normal diet was a purified diet based on the Purina Laboratory Rodent Diet 38057 (Dyets Inc., Bethlehem, PA, USA). The HFD was identical to the ND but supplemented with 350 g fat/kg (300-g lard plus 50-g corn oil) and 1% cholesterol (Research Diets Inc., Bethlehem, PA, USA).

After 10 weeks of high fat diet feeding, mice are sacrificed to collect their blood and internal organs, following a 12-h fast. To analyze plasma lipid levels, blood was drawn into EDTA-coated tubes via cardiac puncture. Plasma was isolated by centrifuging the blood at 2000× *g* for 15 min at 4 °C. The epididymal and visceral fat pads were removed, rinsed with PBS, and weighed. The liver was weighed, photographed, and stored at 80 °C.

All zebrafish experiments were approved by the internal Animal Ethics Committee at CHA University. Embryos and larvae of zebrafish (Danio rerio) were initially obtained from Chungnam National University (Daejeon, Korea).

Larvae were maintained in a 100-mm plate at a density of ~20 larvae per 100 mL, and fed ad libitum with hardboiled egg yolk as a high-fat diet (HFD) once per day, in the presence or absence of SN for 12–15 days (17–20 dpf). SN and vehicle (dimethyl sulfoxide, DMSO) were treated at a concentration of 0.1% (*v*/*v*) in each group plate. Two concentrations of SN (2 and 5 µM) were examined, and CCM (curcumin, 2.5 µM) was used as a positive control. Zebrafish larvae used for imaging analysis were starved for 24 h prior to Nile red staining, to ensure that their digestive tracts were empty.

### 4.10. Quantification of Triglycerides

Triglyceride was evaluated using a triglyceride assay kit (Zen-Bio). Zebrafish was washed with PBS to remove residual medium, and the zebrafish was lysed with RIPA buffer. Triglyceride was digested with Reagent B for 2 h to release hydrolyzed glycerols into the buffer. Diluted hydrolysates were incubated with Reagent A containing peroxidase to produce quinoneimine dye, which shows a absorbance maximum in spectrophotometric detection at 540 nm.

### 4.11. Nile Red Staining, Fluorescence Imaging, and Quantification

For staining of zebrafish, stock solution (1.25 mg/mL, Invitrogen N-1142) of Nile red (Invitrogen N-1142) was prepared in acetone, and store in dark at −20 °C. Briefly, Nile red stock solution was supplemented with water to a final concentration of 50 ng/mL. The fish were incubated in this water for 5–10 min at 28 °C in the dark, after which they were washed with distilled water three times. A few drops of a tricaine (Sigma) stock solution (4 mg/mL, pH 7) were added to induce anesthesia. The fish were mounted in 3% methylcellulose, and the Nile red-stained area was imaged under a fluorescence dissecting microscope (TE300, Nikon, Tokyo, Japan) equipped with a green fluorescent protein (GFP) long-pass filter. Fluorescence images were acquired using a Pixera Penguin 600CL digital camera and the InStudio software (Pixera, Santa Clara, CA, USA).

### 4.12. Biochemical Analysis

Plasma concentrations of total cholesterol, high-density lipoprotein (HDL)-cholesterol, low-density lipoprotein (LDL)-cholesterol, triglyceride, albumin, glucose (Asan Pharmaceutical, Gyeonggi, Korea), and insulin (Shibayagi, Gunma, Japan) were determined enzymatically using commercial kits.

### 4.13. Statistical Analysis

All data are expressed as means ± standard error of the mean of triplicate determinants. All statistical analyses were performed using the SAS (version 9.1, Statistical Analysis Software, Cary, NC, USA). One-way analysis of variance (ANOVA) was used for comparisons among groups. Significant differences between the mean values were assessed using Duncan’s test. *p* values < 0.05 were considered to indicate statistical significance.

## 5. Conclusions

This study showed that SN inhibited lipid accumulation in various models, including the zebrafish. Our findings suggest that SN inhibits early adipogenic processes by suppressing the MCE stage via cell cycle arrest and its regulation of AMPKα, ERK, and Akt signaling pathways contributes to its antiadipogenic effects. Our study provides useful support for algae-based antiadipogenic agents.
